# The NLRP3-Inflammasome in Health and Disease

**DOI:** 10.3390/ijms232113103

**Published:** 2022-10-28

**Authors:** Anna Perri

**Affiliations:** Department of Experimental and Clinical Medicine, University Magna Graecia, 88100 Catanzaro, Italy; anna.perri@unicz.it

The nucleotide-binding domain (NOD)-, leucine-rich repeat (LRR)-, and pyrin domain (PYD)-containing protein 3, NLRP3, is a multiprotein complex belonging to the innate immune system that can be activated by pathogens or danger-associated molecular patterns [1]. The NLRP3 inflammasome is a central proinflammatory signaling complex that, by triggering caspase-1 activation, leads to the maturation of the inflammatory cytokines IL-1β and IL-18. Furthermore, active caspase-1, by cleaving Gasdermin D, can also induce an inflammatory form of programmed cell death, which is known as pyroptosis [2,3]. Of note, chronic low-grade inflammation, characterized by persistent elevated concentrations of circulating pro-inflammatory cytokines, has been associated with the development and progression of a myriad of chronic inflammatory and autoimmune diseases, inducing high impacts on public health, and recent studies demonstrated the crucial contribution of NLRP3 inflammasome activation in sustaining sterile inflammation [4,5]. 

Furthermore, emerging reports on SARS-CoV-2 suggested that NLRP3 inflammasome activation strongly contributes to the formation of the severe inflammatory cytokine storm, resulting in clinical and pathological manifestations of patients infected with COVID-19, especially in those with increased risk [6,7,8].

In view of these evidence, NLRP3 is actually considered as a new drug target with the potential for treating various inflammatory diseases, so small natural and synthetic molecule inhibitors targeting NLRP3 inflammasome signaling are currently developed rapidly, although the efficacies of these agents need further investigation [9].

The Special Issue of the *International Journal of Molecular Science* titled “The NLRP3-Inflammasome in Health and Disease” is devoted to collecting original research and reviews of the literature concerning the role of NLRP3-inflammasome in pathogenetic mechanisms leading to the onset and/or progression of different human chronic diseases, strengthening the observation that NLRP3-inflammasone could represent a new potential therapeutic target. New information has been added to this field by evidence provided by twelve articles, with eight original papers and four narrative reviews.

The original papers described new molecular mechanisms by which low-grade chronic inflammation induced by NRPL3 inflammasome complex activation could contribute to the tissue damage observed in different models of chronic pathological conditions. 

The in vivo study of Burger et al. added new knowledge on the role of NLRP3 activation and IL-1β production in atherosclerosis progression. Interestingly, the authors demonstrated that the infiltrating monocytes into the intima and hypercholesteremia, by triggering NLRP3 inflammasome activation and IL-1β signaling, promoted the vascular smooth muscle cell’s phenotypic switch to macrophages-like cells and the foam cell formation, causing destabilization and the rupture of the atherosclerotic plaques. Overall, the data suggested that they block inflammasome assemblies; thus, IL-1β inhibition production could represent an effective tool for preventing major adverse cardiovascular events [10].

Américo-Da-Silva explored, for the first time, the link between insulin-resistance (IR) and NLRP3 inflammasome activation, demonstrating an increased expression of NLRP3 inflammasome components in the skeletal muscle of obese insulin-resistant animals (HFD) compared to normal control diet mice (NCD). Furthermore, the authors observed, for the first time, that MCC950, a specific inhibitor of NLRP3 inflammasome activation, promoted increased insulin-induced GLUT4 translocation in skeletal muscle fibers from HFD mice and that the inhibitor increased GLUT4 translocation in the absence of insulin in both NCD and HFD muscle fibers. Collectively, the study supports the idea of the skeletal muscle tissue as an active component of inflammatory processes associated with obesity and that the NLRP3 inflammasome might play a physiological role in glucose metabolisms by regulating GLUT4 trafficking in skeletal muscles [11].

Interestingly, Lee et al. provided evidence on the role of NLRP3 inflammasomes in the mechanisms by which glycemic variability is known to be a stronger predictor for microvascular and macrovascular complications than sustained hyperglycemia in patients with diabetes. The authors demonstrated that an acute glucose shift activated the NLRP3 inflammasome to a greater extent than constant high glucose, strengthening the current knowledge on the association between abnormal activation of the NLRP3 and onset and progression of diabetes [12,13,14].

Considerable studies have demonstrated the pathogenetic role of the NLRP3 complex activation in many kidney diseases, occurring both in an inflammasome-dependent and inflammasome-independent manner [15]. In particular, the review of Oliveira et al. extensively described the main studies demonstrating that the activation of NLRP3 inflammasome is a key factor in the pathogenesis and progression of Lupus nephritis, concomitantly highlighting that a significantly higher risk of Lupus nephritis is observed in patients with NLRP3 genetic variants. Finally, the authors discussed the evidence emerging from animal studies on the effects of direct and indirect inhibitors in blocking the NLRP3 pathway and controlling Lupus nephritis activity [16]. 

The in vivo model of rhabdomyolysis-induced acute kidney injuries proposed by Song et al. revealed that myoglobin-induced NLRP3 inflammasome activation strongly contributed to tubular renal injury, elucidating the not yet clear underlying mechanisms. Interestingly, the authors demonstrated that the lack of NLRP3 ameliorated renal tubular injury and, more importantly, that the main factor in decreasing inflammation is the attenuation of inflammatory responses in renal tubular cells rather than renal immune cells [17].

Some studies conducted in patients affected by age-related macular degeneration revealed an abundant amount of NLRP3 in the retina and high levels of IL-1β are in the vitreous body, addressing the pivotal role of NLRP3 machinery activation in the pathogenesis of this degenerative disease [18,19]. The in vitro study of Ranta-Aho et al., conducted in the human retinal pigment epithelium, tested the effects of a novel Hsp90 inhibitor, TAS-116, on NLRP3 activation, showing that the drug prevented the activation of caspase-1, subsequently reducing the release of mature IL-1β. Furthermore, the authors observed a high in vitro therapeutic index, indicating that it should be possible to establish a TAS-116 concentration that has a good anti-inflammatory effect without generating adverse effects [20]. 

The in vivo study of Mae et al. provided new evidence on the role of activated NLRP3 into the pathogenesis of periodontitis. The authors demonstrated that the activation of NLRP3 in macrophages stimulated with dental calculus, a common deposit in periodontitis patients, increased the local production of IL-1β and IL-18 that, in turn, by promoting osteoclastogenic and anti-osteogenic effects, could trigger alveolar bone resorptions [21].

The rat model of reserpine-induced fibromyalgia proposed by D’Amico et al. provided evidence on the involvement of NLRP3 inflammasome activation in the pain states and neuroinflammation of patients affected by fibromyalgia. The authors showed that the pharmacological inhibition of purinergic P2X7 receptor (P2X7R) prevented the NLRP3 inflammasome’s activation and, consequently, the release of IL-1β and IL-18, which are involved in the activation of nociceptors in the spinal cord and brain tissues. The finding emerging from the study suggested that NLRP3 pathway activation could be involved in the heightened sensitivity to pain and contribute to the symptomatology of fibromyalgia [22]. 

The studies investigating NLRP3 inflammasome activation in neuropsychiatric patients suffering from mood disorders showed increased serum levels of NLRP3 complex components, as well as of IL-1β and/or IL-18 cytokine production, further supporting the role of inflammation in psychiatric diseases. The study of Zhou et al. pointed out that the techniques used in these studies are not sufficient for measuring NLRP3 activation. Therefore, the authors developed and validated an assay to measure the intracellular formation of ASC specks in activated PBMC and IL-1β and caspase-1 levels in the periphery over 6 months in adolescents with bipolar and depressive disorders. Despite the small sample size, the results are interesting as, although all patients demonstrated significant increases in ASC specks and extracellular IL-1β production, the authors observed an inter-individual difference in activation magnitudes, suggesting that NLRP3 inhibitors could be candidates for individualized early intervention and combination therapy in patients affected by mood disorders [23].

The review article by Poli et al. highlighted studies investigating the role played by oxidative stress, inflammation, and NLRP3 inflammasome activation in varicocele disease, concomitantly describing some therapeutic antioxidant strategies that have recognized beneficial effects in counteracting the above-reported pathological conditions leading to varicocele-mediated hypofertility. Most evidence reported that the abnormal testicular function and failed spermatogenesis observed in patients affected by varicocele are caused by ROS-driven NLRP3 activation and the overexpression of cytokines that, in turn, further increase ROS production up to and exceeding the availability of antioxidant systems. A variety of antioxidants were assessed for their ability to counteract oxidative stress in the testes, and among them, the most effective antioxidant treatments are non-enzymatic factors such as resveratrol vitamins E and C, coenzyme Q10, and lycopene and therapeutical non-enzymatic drugs, such as Kallikrein, Pentoxifylline, and Cinnoxicam [24]. 

The review of Ryan et al. aimed to highlight findings regarding the molecular mechanisms linking NLRP3 inflammasome activation and post-transplantation complications related to alloimmune injury and rejection. Despite the significant amount of studies, the authors concluded that it is mandatory to better understand how NLRP3 inflammasome activation is spatially and temporally regulated during the acute and chronic post-transplant period, and developing non-invasive biomarker surveillance that could be used as a risk factor stratification for poor clinical outcomes across solid organs is mandatory as well [25].

Effendi et al. summarized the recent evidence focused on NLRP3 inflammasome activation in response to viral infection throughout the development and progression of idiopathic pulmonary fibrosis, the most common chronic-age-related respiratory disease rising from repeated micro-injury of the alveolar epithelium and aberrant epithelial–mesenchymal transitions. The authors provided an overview of the mechanisms through which viral infection triggers the activation of the NLRP3 inflammasome, demonstrating that pyroptosis and IL-1β and IL-18 production strongly contribute to robust inflammation, fibroblast to myofibroblasts differentiation, and the synthesis of the extracellular matrix and epithelial–mesenchymal transition, leading to lung fibrosis. Furthermore, the authors highlighted that some recent evidence support the hypothesis that SARS-CoV-2 might directly activate the NLRP3 inflammasome, leading to cytokine storms and fibrosis and opening a new scenario for the management of COVID-19 [26].

Overall, all studies included in this Special Issue provide an update on the role of NLRP3 inflammasome activations in the pathomechanism of several chronic diseases, highlighting that the NLRP3 signaling pathway is an interesting therapeutic target that may support or even replace traditional therapies in the future.
